# Impact of a procalcitonin-based algorithm on the quality of management of patients with uncomplicated adhesion-related small bowel obstruction assessed by a textbook outcome: a multicenter cluster-randomized open-label controlled trial

**DOI:** 10.1186/s12876-022-02144-w

**Published:** 2022-03-02

**Authors:** Charles Sabbagh, Francois Mauvais, Jean-Jacques Tuech, Christophe Tresallet, Pablo Ortega-Debalon, Muriel Mathonnet, Jeremie H. Lefevre, Zaher Lakkis, David Fuks, Fabrice Muscari, Bernard Dron, Philippe Couderc, Arnaud Alves, Jean-Marc Regimbeau

**Affiliations:** 1 Service de chirurgie digestive, CHU Amiens-Picardie, 1 rond point du Pr Cabrol, 80054 Amiens Cedex 01, France; 2 UR7518 SSPC, universite de Picardie Jules Verne, 80000 Amiens, France; 3 Service de chirurgie digestive, CH Beauvais, Avenue Leon Blum, 60000 Beauvais, France; 4 Service de chirurgie digestive, CHU Rouen, 13 Rue Boucicaut, 76130 Mont Saint Aignan, France; 5 Service de chirurgie digestive, CHU Avicenne, APHP, 125 rue de Stalingrad, 93000 Bobigny, France; 6 Service de chirurgie digestive, 3 Rue du FBG Raines, 21000 Dijon, France; 7 Service de chirurgie digestive, 2 Avenue Martin Luther King, 87042 Limoges, France; 8 Service de chirurgie digestive, CHU Saint Antoine, APHP, 184 rue du faubourg Saint Antoine, 75012 Paris, France; 9 Service de chirurgie digestive, 3 boulevard Alexandre Fleming, 25000 Besançon, France; 10 Service de chirurgie digestive, CHU Cochin, APHP, 27 rue du faubourg Saint-Jacques, 75014 Paris, France; 11 Service de chirurgie digestive, CHU Toulouse, 7 place Lange, 31300 Toulouse, France; 12 Service de chirurgie digestive, CH Saint Quentin, 1 avenue Michel de l’Hospital, 02100 Saint-Quentin, France; 13 Service de chirurgie digestive, CH Pau, 4 boulevard Hauterive, 64000 Pau, France; 14 Service de chirurgie digestive, CHU de Caen, Avenue de la côte de Nacre, 14033 Caen, France

**Keywords:** Procalcitonin, Small bowel, Obstruction

## Abstract

**Background:**

Acute adhesion-related small bowel obstruction (ASBO) is a common digestive emergency, accounting for 1 to 3% of all digestive emergencies. The efficacy of conservative management in this setting is a subject of debate, as it may delay the decision to perform surgery and increase the frequency of bowel resection (e.g., in the presence of bowel necrosis) or, in contrast, prompt an excessive number of unnecessary laparotomies. Thus, the decision to perform surgery is difficult. We propose that the introduction of the procalcitonin (PCT)-based algorithm improves the quality of the management of patients with ASBO by aiding the decision of whether or not to perform surgery.

**Methods:**

This is a 1:1 cluster-randomized clinical trial (use of algorithm: no algorithm) using an independent computer to ensure that investigators cannot interfere with the randomization. Each cluster will correspond to one investigating center. All patients in a center will be managed in the same way. Before randomization, each principal investigator will provide a commitment to participate in the study to avoid the risk of “empty clusters”. The patients included will constitute two parallel arms (use of algorithm versus no algorithm), with no expected crossover between arms. The inclusion criteria are being an adult with uncomplicated acute ASBO (i.e., absence of fever, abdominal pain and distension, nausea and/or vomiting, and the absence of gas and/or stool, in conjunction with a contrast-enhanced CT scan, for patients with previous abdominal surgery) who is able to express consent with a signed written informed consent form. Patients with complicated acute ASBO (strangulation or peritonitis) will be excluded.

**Discussion:**

There is an ongoing debate on the management of uncomplicated ASBO. The main points are to avoid a surgery if it is unnecessary and to avoid delayed surgery if it is necessary. Currently, there are no robust criteria to objectively determine the failure of non-surgical treatment or to establish the indications for surgery in acute ASBO. Our team proposes the use of procalcitonin (PCT) to help distinguish patients for whom conservative management is likely to be successful from those for whom surgical management is required. The results from a randomized control trial could help in the selection of patients through clear inclusion and exclusion criteria and simplify or clarify the management algorithm. In conclusion, PCT may be useful in evaluating the proper strategy for ASBO.

*Trial registration* The trial is registered at clinical trials under the reference: NCT03905239

## Background

Acute adhesion-related small bowel obstruction (ASBO) is a common digestive emergency, accounting for 1 to 3% of all digestive emergencies [1, 2]. It is associated with a mortality rate of between 2 and 8%, although this figure may be as high as 25% when surgical treatment is delayed [3–6]. According to a study based on the medical records of the Scottish National Health Service over a 10-year period, 5.5% (n = 1169) of the 21,347 patients admitted for ASBO underwent surgery [7]. The associated costs are non-negligible (approximately $9000 for cases with conservative management and between $30,000 and $40,000 for cases requiring surgery) [8]. In 2013, the working group on ASBO of the World Society of Emergency Surgery suggested two distinct approaches for the management of acute ASBO [9]. Conservative (i.e., non-surgical) management can be initiated when there are “no signs of strangulation or peritonitis or a history of persistent vomiting or combination of CT scan signs (free fluid, mesenteric edema, small bowel feces signs, devascularized bowel)”, whereas surgical management (with or without bowel resection) must be initiated before or during conservative management in the event of “free intraperitoneal fluid, mesenteric edema, the presence of small bowel feces signs by CT, a history of vomiting, severe abdominal pain (VAS > 4), abdominal guarding, elevated WCC, and devascularized bowel by CT” [9]. Conservative management includes the use of a nasogastric tube (NGT), intravenous administration of fluids, and clinical and biochemical monitoring for 24 to 72 h [9]. However, the efficacy of conservative management in this setting is a subject of debate, as it may delay the decision to perform surgery and increase the frequency of bowel resection (e.g., in the presence of bowel necrosis) or, in contrast, prompt an excessive number of unnecessary laparotomies. Surgical management consists of adhesiolysis and (in some cases) bowel resection.

Currently, there are no robust criteria to objectively determine the failure of non-surgical treatment or establish the indications for surgery in acute ASBO. Gastrografin has been evaluated in this setting but several meta-analyses failed to show any benefit of gastrografin [10].

As no criterion can be used to identify the failure of conservative management or propose surgery as initial management, biomarkers, such as procalcitonin (PCT), have been investigated in this setting. PCT has been described as a marker of infection and inflammation. We previously tested PCT as a discriminant factor for necrosis in mesenteric infarction, with a cutoff of 2.4 ng/ml [11]. Then, our team proposed the use of PCT to help distinguish patients for whom conservative management is likely to be successful from those for whom surgical management is required. Cutoffs of 0.2 µg/L (for failure of conservative management) and 0.6 µg/L (for need for surgery) accurately identified more than 80% of patients [12]. These cutoffs and data were confirmed in a second independent cohort and were then used to propose an algorithm for the management of patients with ASBO. In this single-center, retrospective, case–control study, we showed that the introduction of this algorithm to patient management reduced i/ the time to surgery, with no increase in the surgical management rate, and ii/ the length of stay (with a 2-day difference) [13].

We propose that introduction of the PCT-based algorithm will improve the quality of management of patients with ASBO. We expect that the arm combining standard management with the PCT-based algorithm will be superior to the arm with standard management alone in terms of the success rate for patients with ASBO. If the algorithm is shown to improve patient management and is cost-effective, it could be proposed in routine clinical practice.

## Methods/design

### The aim, design, and setting of the study

#### The aim

The primary objective of the study is to assess the impact of a PCT-based algorithm on the textbook outcome at postoperative day 90. The secondary objectives are to compare short-term and long-term recurrence rates, quantify and compare patient satisfaction, compare morbidity according to the type of management, compare the length of stay, propose other markers of ischemia (copeptin, proadrenomedullin) to reinforce the value of PCT in the early stages of ASBO, and perform a cost-effectiveness analysis.

#### The design

This is a 1:1 cluster-randomized clinical trial (use of algorithm: no algorithm) using an independent computer to ensure that investigators cannot interfere with the randomization. Each cluster corresponds to one investigating center. Randomization is center-based, i.e., all patients will be managed in the same way. Before randomization, each principal investigator will provide a commitment to participate in the study to avoid the risk of “empty clusters”. Randomization will be performed using computer-generated random numbers by the trial methodologist before patient recruitment.

#### Setting

The trial will be open in 16 centers. Each of the 16 centers will be assigned to one arm (with or without the PCT algorithm) to avoid contamination between the two arms.

### Characteristics of the participants

Patients are eligible for inclusion if they are over 18 years of age, have an acute ASBO (abdominal pain and distension, nausea and/or vomiting, the absence of gas and/or stool, in conjunction with contrast-enhanced CT scan showing no other cause for obstruction for patients with previous abdominal surgery), without signs of complications (strangulation, peritonitis), are able to express consent, signed the written consent form, and are covered by national health insurance. They are not eligible for inclusion if they have had no previous abdominal surgery, if they have signs of strangulation, bowel ischemia or strangulation, if they have an obstruction within four weeks following a previous surgery, an ongoing or history of bowel cancer, an ongoing or history of inflammatory bowel disease, a history of abdominal radiotherapy, an active infection, or a contraindication to contrast-enhanced CT.

The target population is defined as patients requiring management for CT-scan-confirmed uncomplicated adhesion-related small bowel obstruction. These patients will be recruited by public hospital emergency department consultations (university and non-university hospitals).

### Processes, interventions, and comparisons

**Fig. 1:**
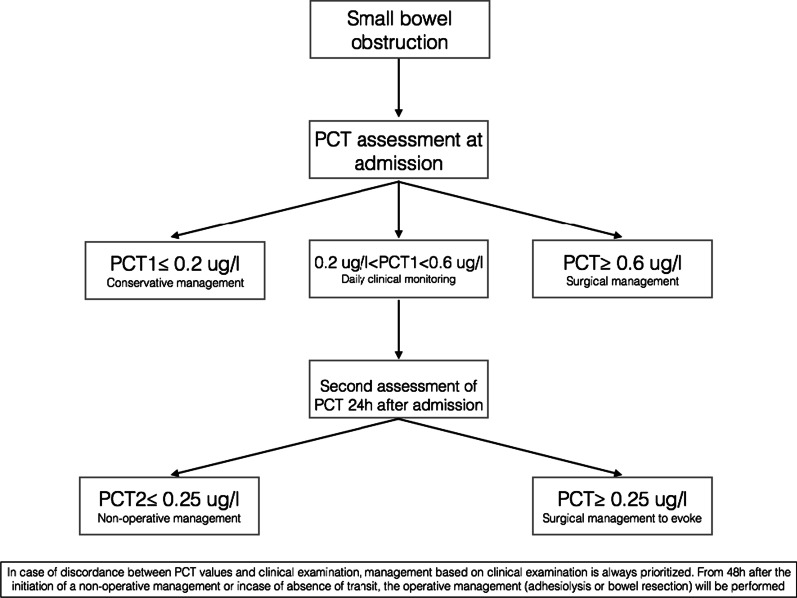
Synopsis of the study

In the algorithm group, patient management will be based on clinical examination and PCT assessment. PCT will be assayed on admission. If PCT is between 0.2 and 0.6 µg/L, a second assay will be performed 24 h after admission (Fig. 1). From 48 h after the initiation of conservative management or in the absence of bowel function, operative management (adhesiolysis or bowel resection) will be performed. In the event of discordance between PCT values and the clinical examination i.e., the presence of persistent ASBO or signs of complications, management will be based on the clinical examination. Conservative management will be continued for 48 h in the absence of signs of bowel ischemia and the presence of bowel function.

In the control group, patient management will be based on clinical examination. Gastrografin will not be used in the control arm, as data from a recent randomized clinical trial that included 242 patients (ABOD study [10]), combined with the results of a meta-analysis performed in 2015 that included 990 patients, failed to demonstrate the value of gastrografin to reduce the surgery rate and length of stay. Gastrografin may therefore no longer be indicated in this setting. The use of gastrografin could also introduce a bias in the interpretation of the outcome, as the outcomes could be considered to be related to gastrografin and not exclusively to the algorithm. Conservative management will be continued for 48 h in the absence of signs of bowel ischemia and the presence of bowel function.

From 48 h after the initiation of conservative management or in absence of bowel function, operative management (adhesiolysis or bowel resection) will be performed.

There is no blinding procedure, as blinding is not possible/necessary because each center will be randomized to one study arm and all patients in each center will be managed according to the same strategy.

### Outcomes

The primary endpoint will be the proportion of patients achieving the textbook outcome, defined as patients either appropriately undergoing surgery (ischemia confirmed at operation ± resection) or appropriately managed conservatively (no need for surgery) with no major postoperative complications (Clavien–Dindo ≥ 3) and a medical length of stay < 5 days (defined as the time at which the patient is medically eligible for discharge), with no postoperative consultation, rehospitalization, or reoperation within 90 days after randomization. The textbook outcome is defined as that proposed by Kolfschoten [14] for colon cancer resection. The primary endpoint will be analyzed by an open-label design. The centers will record all cases of (i) no readmission, (ii) no unplanned consultation for nausea, vomiting, and/or the absence of gas and/or stool, (iii) no hospitalization, (iv) no reoperation, and (v) no recurrence that could occur after the patient’s discharge and before the two visits scheduled in the protocol (1 and 3 months). If the quality of the completion of the primary endpoint is not sufficient after monitoring of the first-recruited patients, the adjudication committee will be contacted.

The secondary endpoints are the 1-, 3-, 6-, 9-, 12-month recurrence rates, defined as a new episode of adhesion-related small bowel obstruction, the EVAN G score to evaluate patient satisfaction at postoperative month 1 [15], the Clavien and CCI scores to assess morbidity evaluated after the patient’s discharge at postoperative month 1, the hospital length of stay, defined as the interval between admission to the emergency department and discharge from the ward, and the cumulative length of stay, defined as the total number of days of hospitalization related to ASBO at postoperative month 12.

### Sample size

According to Williams, Ellozy, Fraser et al., 60% of patients were successfully managed without the algorithm. Ryan also reported that 62% of patients were not readmitted or hospitalized for ASBO at postoperative day 90. With our local data showing successful management with the algorithm in 78% of patients and assuming a 62% success rate without the algorithm, with a two-sided alpha risk of 5%, a power of 80%, an intra-class correlation coefficient of 0.015, 16 anticipated clusters (centers), a coefficient of variation of 0.15 for cluster size, 372 patients would need to be recruited. By assuming 5% of patients lost to follow-up, 414 patients need to be enrolled.

### Study calendar

The planned duration of inclusion is 24 months and the duration of participation for each participant is three months. The planned total duration of the study is 27 months. This is the version 1 of the protocol (17/11/2021).

### Randomization procedure

After obtaining consent from potential participants, randomization will be performed using computer-generated random numbers by the trial methodologist before patient recruitment. Each of the sixteen centers will be assigned to one arm (with or without the PCT algorithm) in order to avoid contamination between the two arms. In the case of typing errors or missing data, a message will indicate the corrections to be made to allow randomization of the patient.

### Stopping rules

The patients can withdraw their consent and leave the study at any time, for any reason. In case of anticipated stop, the investigator must precise the reason as complete as possible. The investigator can terminate the patient’s participation for any reason in the patient’s best interests, especially in the presence of adverse events. The study can be stopped in the case of unexpected serious adverse events.

In the event of premature discontinuation of the study, exclusion from the study, study drop-out or major protocol deviation, patients will be followed as usual.

### Ethics

The study received an agreement from the Comité de protection des personnes under the number 2017-A00613-50/SI: 18.09.12.65718 on the 29th of November 2018.

### Access to data

The sponsor is responsible for obtaining the agreement of all parties involved in the study to ensure direct access to all study sites, source data, source documents, and reports to enable the sponsor to control data quality and perform an audit. Investigators will provide access to documents and individual data strictly required for monitoring, quality control and audit of the biomedical study to authorized persons, in accordance with current statutory and regulatory provisions (Articles L.1121-3 and R.5121-13 of the French Public Health Code). Any original document or object establishing the existence or accuracy of a data point or information recorded during the study is defined as a source document (medical file, original copy of clinical, laboratory test and imaging results, examination reports, etc.). In accordance with current statutory provisions (Articles L.1121-3 and R.5121-13 of the French Public Health Code), persons with direct access to source data will take every necessary precaution to ensure the confidentiality of information relating to investigational medicinal products, studies, participants, especially concerning the identity of these subjects, as well as the results obtained. These persons, like the investigators themselves, are bound by professional secrecy. All data concerning participants collected during or at the end of the study will be coded by the investigators (or any other specialists involved) before being sent to the sponsor. The name and address of study participants will never be directly visible. Only personnel participating in the study will have access to the patients’ data.

Confidentiality of data will be ensured by coding patient information based on the following data:

- Their initials: the first letter of the last name and the first letter of the first name,
- A 5-digit study number: a 2-digit number corresponding to the study center number, followed by a 3-digit number from 001 to 999 attributed by order of inclusion in the study.

The sponsor will ensure that each study participant has given his/her written consent for access to his/her personal data as strictly required for quality control of the study.

### Protocol amendment

Any substantial modification, i.e. any modification likely to have a significant impact on the safety of study participants, the conditions of validity and results of the study, the quality and safety of investigational products, the interpretation of scientific documents that support the study or the study procedures, must be described in a written amendment that will be submitted to the sponsor, and must be approved by the ethics committee (CPP) and authorized by ANSM before implementation.

Non-substantial modifications, i.e. those with no significant impact on any aspect of the study, will be notified to the CPP for information purposes.

All amendments must be validated by the sponsor, and by all parties involved in the study concerned by the modification, before submission to the CPP and ANSM. This validation may require a meeting of the scientific committee and/or independent monitoring committee.

All investigators participating in the study must be informed about all protocol amendments. The investigators must undertake to comply with the contents of all protocol amendments.

Any amendment that modifies patient care or the benefits, risks and constraints of the study will require a new patient information sheet and a new informed consent form, according to the aforementioned procedure.

### Dissemination policy

Analysis of data provided by the investigating centers is performed by Amiens University Hospital. This analysis will result in a written report that will be submitted to the sponsor, who will then submit the report to the ethics committee (Comité de Protection des Personnes) and the competent authority.

All written or oral communication of study results must receive prior approval from the coordinating investigator and, when applicable, the committees formed for the study.

Publications of the principal results will cite the name of the sponsor, all investigators who included or followed participants in the study, methodologists, biostatisticians, and data managers who participated in the study, the members of the study committee(s), and, if applicable, the participation of a drug company/the source of funding. International requirements for writing and publication will be taken into account (The Uniform Requirements for Manuscripts, ICMJE, April 2010).

## Discussion

The management of complicated ASBO is straightforward, as surgery is required. There is, however, an ongoing debate concerning the surgical management of uncomplicated ASBO [16]. The main points are to avoid surgery if it is unnecessary and to avoid delaying surgery if it is necessary. A recent propensity score analysis of 27,904 patients showed a lower risk of recurrence than with conservative management (13.0% vs 21.3%, p < 0.01). Another study of 6,191 patients confirmed these outcomes (19% vs 25.6%, p < 0.005). However the authors noted a higher mortality rate in the operative group (3.7% vs 2.6%, p = 0.025) [17]. The debate is still ongoing and conservative treatment remains the standard of care whenever possible.

Currently, there are no robust criteria for objectively determining the failure of non-surgical treatment or establishing the indications for surgery in acute ASBO. Soluble contrast medium (such as gastrografin) is still used in a number of centers as a diagnostic test for the resolution of small bowel obstruction when it is observed in the cecum by abdominal X-ray. The therapeutic effect of water-soluble contrast medium is due to a side effect based on the stimulation of bowel peristalsis [10]. However, the efficacy of water-soluble contrast medium in this setting is also subject to debate, as data from a recent randomized clinical trial that included 242 patients (ABOD study), combined with a meta-analysis in 2015 that included 990 patients, failed to demonstrate any value of gastrografin to reduce the surgery rate and length of stay [11, 12]. In the ABOD study, the operative intervention rate was 24% in the gastrografin arm and 20% in the control arm (p = NS) and the bowel resection rate was 8% in the gastrografin arm and 4% in the control arm (p = NS). These results, combined with those of a meta-analysis based on 10 published studies, suggest that gastrografin did not decrease either the operative intervention rate (26% in the gastrografin arm versus 21% in the control arm) or the number of days from initial CT to discharge (3.5 versus 3.5, p = NS for both) [12].

The results from a randomized control trial could help in the selection of patients by clear inclusion and exclusion criteria and simplify or clarify the management algorithm [10]. The aim of this randomized control trial is to simplify the algorithm to avoid unnecessary waiting for patients who need surgery. A PCT level > 0.6 ug/l at admission or > 0.25 ug/l 24 h after admission is highly suggestive of bowel ischemia. On the other hand, a procalcitonin level < 0.2 ug/l may be reassuring, as it reveals no ischemia and might encourage the implementation of improved recovery programs. This could have an impact on the quality of life of the patients, the length of stay, and the medico-economic criteria.

No criterion can be currently used to identify the failure of conservative management or to propose surgery as initial management. Thus, biomarkers, such as PCT have been investigated in this setting. PCT has been described as a marker of infection and inflammation. We tested PCT as a discriminant factor for necrosis in mesenteric infarction with a cutoff of 2.4 ng/ml [13]. Then, our team proposed the use of PCT, to help distinguish patients in whom conservative management is likely to be successful from those in whom surgical management was mandatory. Cutoffs of 0.2 µg/L (for failure of conservative management) and 0.6 µg/L (for need for surgery) accurately identified more than 80% of patients [14]. These cutoffs and data were confirmed in a second independent cohort, and were then used to propose an algorithm for the management of patients with ASBO. In this single-center, retrospective, case–control study, we showed that introduction of this algorithm into patient management reduced i/ the time to surgery with no increase of the surgical management rate; ii/ the length of stay (with a 2-day difference) [15]. We propose the hypothesis that introduction of the PCT-based algorithm improves the quality of management of patients with ASBO.

We hypothesize that introduction of the PCT-based algorithm will improve the quality of management of patients with ASBO.

Several points in the methodology of the present study merit discussion. The first is the method of randomization by center. We decided that each center would follow a homogeneous flow-chart for patient management, as determined by the randomization, to reduce the evaluation bias and/or attrition bias that could potentially occur if the center changed its management of patients during the study. Furthermore, as the management of ASBO requires an emergency procedure, it would be difficult to randomize individual patients, which could result in a high rate of contamination between arms.

In conclusion, PCT may be useful in helping to determine the correct strategy in uncomplicated ASBO.

## Data Availability

Dataset generated or analysed during the study are available in a protected database under the responsibility of DRCI-projets@chu-amiens.fr.

## Abbreviations

PCT: Procalcitonin
ASBO: Acute adhesion-related small bowel obstruction
CT scan: Computed tomography scan
EVAN G: Evaluation du vécu de l’anesthésie générale, means Evalaution of the satisfaction of the perioperative period
CCI: Charlson Comorbidity Index
